# Impact of a Transparent Easy Nudge-Based Notification on the Follow-Up Examination Behaviors in Japanese Workers: A Controlled Experiment

**DOI:** 10.31662/jmaj.2025-0570

**Published:** 2026-04-03

**Authors:** Masaki Takebayashi, Tatsuya Koyama, Yudai Kaneda, Yuri Mizota, Hirohide Shibutani, Mira Namba, Kouichi Tamura, Yasuo Terauchi, Hirokazu Okada

**Affiliations:** 1 Graduate School of Health Sciences, Aomori University of Health and Welfare, Aomori, Japan; 2 Faculty of Sociology, Aomori University, Aomori, Japan; 3 Center for Nutritional Epidemiology and Policy Research, National Institute of Health and Nutrition, Settsu, Japan; 4 Clinical Training Center, Jyoban Hospital of Tokiwa Foundation, Fukushima, Japan; 5 Graduate School of Public Health, Shizuoka Graduate University of Public Health, Shizuoka, Japan; 6 School of Medicine, Keio University, Tokyo, Japan; 7 Department of Medical Science and Cardiorenal Medicine, Yokohama City University Graduate School of Medicine, Yokohama, Japan; 8 Yokohama City University Medical Center, Yokohama, Japan; 9 Department of Endocrinology and Metabolism, Graduate School of Medicine, Yokohama City University, Yokohama, Japan; 10 Department of Nephrology, Saitama Medical University, Saitama, Japan

**Keywords:** behavioral economics, follow-up examinations, transparent nudges, early detection, noncommunicable diseases, attendance rate, occupational health

## Introduction

The World Health Organization has emphasized that detection, screening, and treatment are key components in addressing noncommunicable diseases ^(1)^. However, they remain a challenge even in countries with advanced public health systems, such as Japan. Annual health checkups are mandatory for workers in Japan; however, follow-up examinations for individuals with abnormal findings are not mandatory. Consequently, less than 50% of Japanese people with abnormal findings attend follow-up examinations, leaving high-risk individuals undiagnosed and untreated ^(2)^. One possible explanation for this noncompliance is that attending follow-up examinations constitutes an intertemporal choice, where present efforts yield future benefits, which many individuals tend to procrastinate ^(3)^.

Nudges―choice architectures that alter people’s behaviors predictably without forbidding any options or significantly changing their economic incentives―are useful for preventing such procrastination ^(3)^. The first principle of the nudge framework is “making things easy (Easy nudge),” which includes improving readability (i.e., enhancing cognitive ease) and providing specific instructions (i.e., supporting decision structure) ^(4)^. A meta-analysis has demonstrated the effectiveness of Easy nudges, and the Japanese government has recommended their use to promote participation in health checkups ^(5), (6)^.

Despite these benefits, empirical applications of Easy nudges to promote follow-up examinations in occupational health remain scarce. Such hesitancy may stem from concerns that nudges are effective only under covert conditions, which can pose ethical dilemmas for occupational health professionals ^(7)^.

Transparent disclosure of Easy nudges―for instance, explicitly stating that “Easy nudges are being implemented to promote behavioral change”―may help mitigate these concerns. To our knowledge, the impact of transparent Easy nudges on follow-up examination behaviors has not yet been empirically demonstrated. Our previous online study demonstrated that a notification promoting follow-up examinations using Easy nudges received positive evaluations from occupational health staff and workers in Japan ^(8)^. However, that study neither employed transparent Easy nudges nor evaluated participants’ attendance rates ^(8)^. Therefore, it is necessary to evaluate transparent Easy nudges in real-world settings. The acceptance of transparent nudges may be limited since Japan is internationally categorized as a “nudge-cautious” country ^(9)^. Evaluating the acceptability of transparent nudges in Japan may support their applicability in other countries.

This study aimed to test the hypothesis that workers with abnormal findings were more likely to attend follow-up examinations after receiving a notification incorporating transparent Easy nudges.

## Materials and Methods

### Research design

A controlled experiment was conducted in two parallel groups during the first half of the 2024 fiscal year (April-September 2024).

### Settings

Five centers of a Japanese transportation company located in the same region were selected. Random allocation was not feasible due to organizational constraints; therefore, a matched allocation approach was used to minimize baseline differences between groups. Specifically, centers were first compared based on predefined characteristics, including the proportion of workers who had attended follow-up examinations in the previous year, geographic location (all centers were in the prefectural capital), organizational size, and predominant job types. The centers were grouped to closely match these characteristics, with two centers assigned to the nudge group and the remaining three assigned to the control group, thereby ensuring balance between groups.

The inclusion criterion was workers who had abnormal findings in the regular health checkups conducted in 2024. The exclusion criteria were industrial health staff and workers who refused to participate in the study. The interventions were implemented using notifications that instructed recipients to independently make a doctor’s appointment and report their appointment status within 3 weeks. These notifications were enclosed with the results of the annual health checkups, and the workers received them after June 2024. An external behavioral economist confirmed the notification design.

### Control group

**Figure 1.**
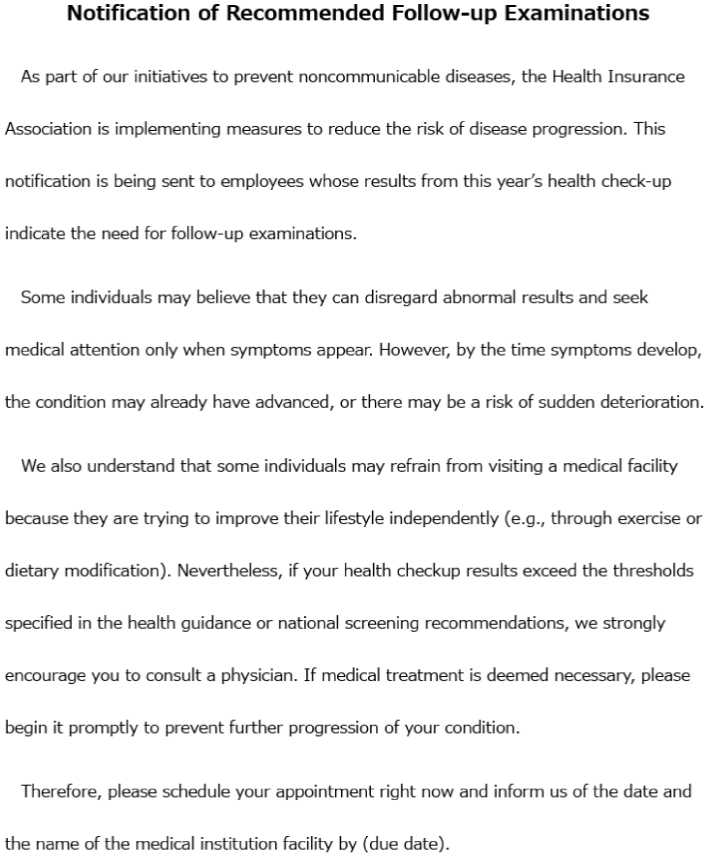
A notification for the control group.

The notification for the control group was based on that previously used by the company (Figure 1). Workers needed to search for appropriate medical departments themselves.

### Nudge group

**Figure 2.**
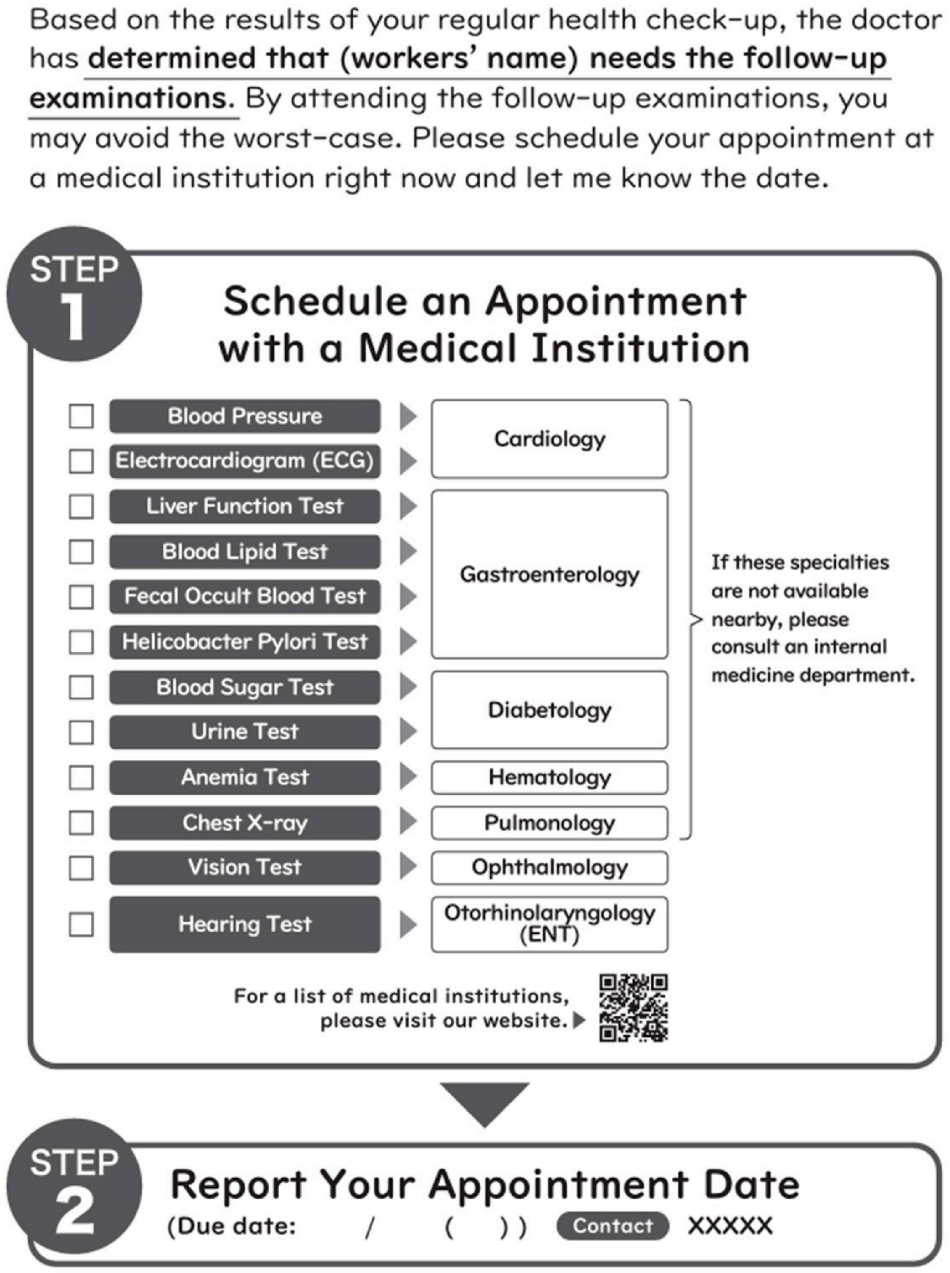
A notification for the nudge group. The notification for the nudge group was adapted from that of the control group based on the Easy nudge concept. It incorporated two key strategies: reducing health information to improve readability and specifying the recommended medical departments to simplify decision-making.

The notification for the nudge group (Figure 2) was developed from that of the control group based on the concept of Easy nudges. To improve readability, health information, which many workers already appeared to know, was reduced. To simplify the decision-making process, the medical departments to be visited were explicitly specified ^(4), (8)^.

### Outcomes and statistical analysis

The primary outcomes were reported appointment status and attendance at follow-up examinations within 3 weeks of notification.

The sample size of 88 participants (44 in each group) was calculated using G*Power (version 3.1.9.4; Heinrich-Heine-University, Düsseldorf, Germany), assuming a power of 80%, an α error of 5%, and an effect size of 30%. Continuous variables were analyzed using t-tests, and categorical variables were analyzed using χ^2^ or Fisher’s exact tests. All baseline characteristics were included as covariates in the adjusted analysis using multivariable regression models. SPSS software (version 28, IBM, Tokyo, Japan) was utilized for data analysis. Statistical significance was set at p < 0.05 (two-tailed).

### Disclosure of nudges and ethics

In May 2024, participants in the control group were informed of the importance of follow-up examinations through the company intranet, whereas participants in the nudge group were informed of the use and purpose of Easy nudges.

This study was approved by the Research Ethics Committee of Aomori University (Approval No. 06-2023). All methods were conducted in accordance with the Declaration of Helsinki and adhered to the Good Clinical Practice guidelines. All workers were informed that data were collected for research purposes and that anyone could express disagreement with participation. After the completion of the study, participants were allowed to review both notifications.

## Results

**Figure 3.**
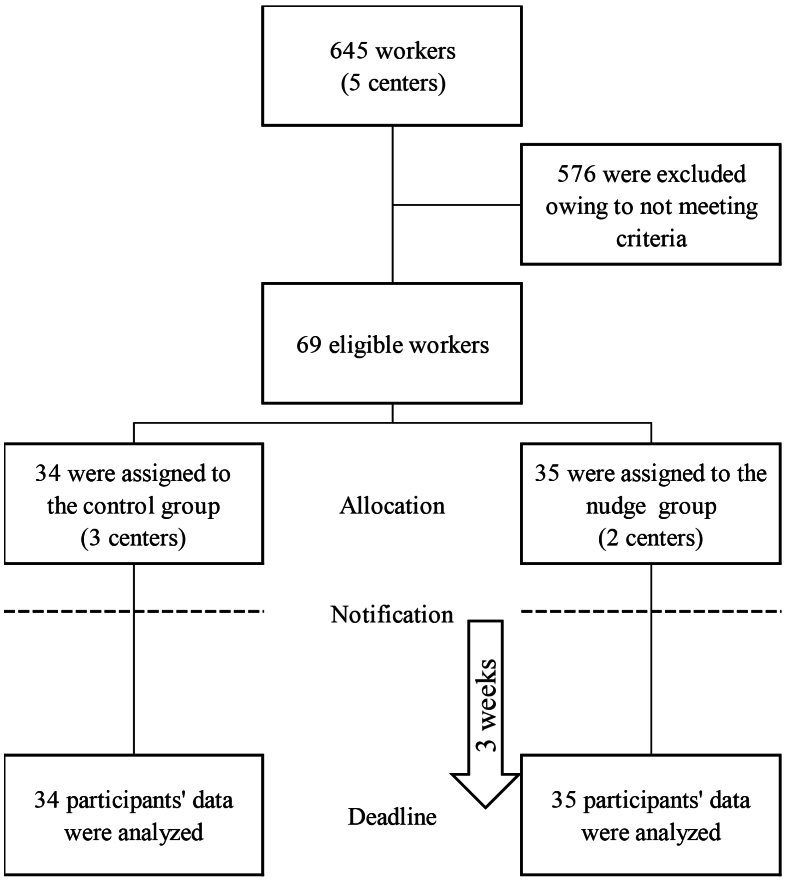
Flow diagram of participants.

All workers with abnormal findings during annual health checkups (34 in the control group and 35 in the nudge group) were included in the analysis (Figure 3). No significant differences were observed in baseline characteristics between the groups (Table 1). Regarding outcomes, 11.8% and 62.9% of participants in the control and nudge groups, respectively, reported their appointment status (p < 0.001). Follow-up examinations were attended by 20.6% and 48.6% of participants, respectively (p = 0.013; Table 2). No adverse events were observed.

**Table 1.** Basic Attributes of Participants.

Items		Control group (N = 34)		Nudge group (N = 35)		p-Value
		n, mean	%, ±SD	n, mean	%, ±SD	
Sex	Men	31	91.2%	26	74.3%	0.110
	Women	3	8.8%	9	25.7%	
Age, years		50.9	±8.6	48.3	±9.6	0.246
Occupation type	Office workers	2	5.9%	2	5.7%	1.000
	Drivers	32	94.1%	33	94.3%	
Workers with abnormal findings in 2023		31	91.2%	32	91.4%	1.000
The number of abnormal findings in 2024		1.1	±0.4	1.3	±0.5	0.113

A t-test was conducted for age, and Fisher’s exact test was used for categorical variables. SD: standard deviation.

**Table 2.** Participant Behavior after Receiving the Notification.

Items	Control group (N = 34)		Nudge group (N = 35)		p-Value
	n	%	n	%	
Workers who reported appointment status	4	11.8%	22	62.9%	<0.001
Workers who attended follow-up examinations	7	20.6%	17	48.6%	0.013
Workers who did neither	27	79.4%	6	37.1%	<0.001

Fisher’s exact test or χ^2^ test was conducted as appropriate for each comparison. p-Values were adjusted for baseline characteristics using analysis of covariance. Owing to the small sample size, clustering adjustments for outcomes were unfeasible.

## Discussion

The nudge group showed substantially higher reporting of appointment status (> 5-fold) and attendance at follow-up examination (> 2-fold) compared with the control group.

The nudge group notification may represent a useful option, given that nudge-based approaches recommended by the Japanese government to promote health checkups have not incorporated notifications specifying medical departments ^(6)^. The nudge group notification incorporated two Easy nudges: improving readability and providing specific instructions. Previous studies have suggested that structuring decisions (e.g., specific instructions) exerts a stronger effect than enhancing readability alone ^(5), (10)^. This suggests that explicit specification of medical departments may have substantially contributed to the observed impacts. This notification could also be beneficial for occupational health staff, as it reduces efforts to answer inquiries such as “Which department should I visit?” from workers. It required only minor wording modifications and could provide benefit at a low cost, meeting the needs of occupational health staff.

These findings are encouraging, but they could not fully support the hypothesis due to some limitations. First, this study was not a randomized controlled trial. Second, we were unable to obtain certain data related to potential cluster-level biases, such as organizational culture, the involvement of occupational health staff, and workload, which may have been influenced by unmeasured confounding factors. Third, the follow-up period was limited to 3 weeks because of constraints at this company. Owing to external factors, such as hospital congestion, some participants may have been unable to schedule appointments within this 3-week period. Given that previous studies have used follow-up periods of 6 or 12 months, future research may consider adopting longer follow-up durations ^(11)^. Fourth, the analyzed sets in both groups were smaller than the estimated sample sizes, which might reduce statistical power. Fifth, the novelty of the intranet announcement to the nudge group, which introduced the Easy nudge, may have had a greater impact on participants than the usual health information received by the control group. This difference in message novelty may have contributed to the observed results. Sixth, we could not assess participants’ understanding regarding the transparency of Easy nudges, and some participants may not have fully understood it. Further studies that address these limitations are necessary.

## Article Information

### Acknowledgments

The authors thank Nippon Express Cash Logistics Co., Ltd. for their support. The development of this manuscript was supported by Katsuhito Ihara, MD, MPH, and Nippon Boehringer Ingelheim.

### Author Contributions

Conceptualization: Masaki Takebayashi and Yuri Mizota. Data curation: Masaki Takebayashi and Tatsuya Koyama. Formal analysis: Tatsuya Koyama. Writing - original draft: Masaki Takebayashi. Writing - review and editing: Masaki Takebayashi, Yudai Kaneda, and Mira Namba. Supervision: Hirohide Shibutani, Hirokazu Okada, Yasuo Terauchi, and Kouichi Tamura. All authors approved the final version of the manuscript.

### Conflicts of Interest

The authors met the criteria for authorship recommended by the ICMJE. The authors did not receive any funding related to the development of this manuscript. This study was supported and funded by Nippon Boehringer Ingelheim Co., Ltd. and Eli Lilly Japan K.K., with Masaki Takebayashi and Yuri Mizota receiving funding from the research team via Ozma PR Inc. Masaki Takebayashi also received consultancy fees from Nippon Boehringer Ingelheim Co., Ltd. and Ozma PR Inc. outside of this project. Yasuo Terauchi received consulting or lecture fees from Astellas Pharma Inc., AstraZeneca K.K., Eli Lilly Japan K.K., Kowa Company, Ltd., Mitsubishi Tanabe Pharma Corporation, MSD K.K., Nippon Boehringer Ingelheim Co., Ltd., Novo Nordisk Pharma Ltd., Sanofi K.K., and Sumitomo Pharma Co., Ltd. and grants from Nippon Boehringer Ingelheim Co., Ltd., Eli Lilly Japan K.K., and Sumitomo Pharma Co., Ltd. KT received honoraria or lecture fees from Nippon Boehringer Ingelheim, AstraZeneca, Novartis, Bayer Yakuhin, Otsuka, Fuji, Kyowa Kirin, Ono, Sanwa Kagaku, Mochida, Kowa, Eli Lilly Japan, and Novo Nordisk; commissioned clinical trials, contract research, and joint research funding from AstraZeneca, Bayer Yakuhin, Novartis, Chinook, Otsuka, Novo Nordisk, Terumo Corporation, Viatris, and Kowa; scholarship donations from Nippon Boehringer Ingelheim, Otsuka, Bayer Yakuhin, and Mochida.

### Approval Code Issued by the Institutional Review Board (IRB) and the Name of the Institution(S) That Granted the Approval

This study was approved by the Aomori University Research Ethics Committee (Approval No. 06-2023). All methods were conducted in accordance with the Declaration of Helsinki and adhered to the Good Clinical Practice guidelines.

### Data Access

Due to confidentiality restrictions, the datasets analyzed in the current study are not publicly available. These datasets are available from the corresponding author upon request.
